# Roadside experiences of parents of children with developmental coordination disorder and/or attention deficit hyperactivity disorder

**DOI:** 10.3389/fnhum.2024.1339043

**Published:** 2024-04-10

**Authors:** Rayan Falemban, Kate Wilmut, Heather Hurst, Catherine Purcell

**Affiliations:** ^1^Department of Physical Therapy, Faculty of Applied Medical Sciences, Umm Al-Qura University,, Makkah, Saudi Arabia; ^2^School of Healthcare Sciences, College of Biomedical and Life Sciences, Cardiff University, Cardiff, United Kingdom; ^3^Centre for Psychological Research, Faculty of Health and Life Sciences, Oxford Brookes University, Oxford, United Kingdom

**Keywords:** pedestrians, attention deficit hyperactivity disorder, developmental coordination disorder, road crossing, risky behavior, child safety, executive function, parental concerns

## Abstract

**Introduction:**

Pedestrians are a vulnerable group at the roadside and previous research has identified that children with DCD and ADHD are at a heightened risk of pedestrian injuries. Despite this, limited research has explored parental perspectives of the pedestrian risks faced by children with DCD and/or ADHD. Understanding parents’ perspectives provides a unique insight into the challenges children face every day and the concerns that parents perceive regarding their children’s safety as pedestrians. Therefore, the aim of this study was to explore parents’ perspectives of the pedestrian risks faced by their children with DCD and/or ADHD.

**Methods:**

Semi-structured interviews were conducted with 14 parents of primary school and early secondary school aged children with age range 7–17. The participants were divided into three groups based on their children’s conditions: DCD group (10–17 years, *n* = 3), ADHD group (7–13 years, *n* = 5), and co-occurring group (7–16 years, *n* = 6). All parents confirmed an existing diagnosis and completed the SNAP-IV and DCDQ as screening tools. The interviews explored parents’ perspectives regarding their children’s pedestrian behaviors, parents’ concerns and preventative measures taken to improve the pedestrian safety of their children with DCD and/or ADHD. Reflexive thematic analysis was undertaken to analyze the interviews, from which three themes were developed.

**Results:**

The first theme related to the challenges experienced by children at the roadside; parents emphasized the significance of structured and controlled pedestrian crossing sites, underlining their preference for designated crossings as safer options due to their heightened perceptions of risk associated with other road-crossing locations. The second theme: parental concerns and influences on children’s road safety referred to their children’s performance and safety at the roadside, leading to increased monitoring and a more protective approach to road crossing. The third theme: road safety education related to various strategies parents implemented to mitigate risks, while balancing independence and prioritizing their safety.

**Discussion:**

While there were commonalities in the challenges faced by children with DCD and/or ADHD at the roadside, there were also notable differences. Parents of children with DCD discussed challenges with spatial awareness and motor skills, whereas parents of children with ADHD discussed challenges with impulsivity and inattention. Parents of children with co-occurring DCD and ADHD described a complex interplay of these challenges. It is evident from the interviews that children with DCD and/or ADHD require a distinct approach to develop their pedestrian skills effectively and parents reported specific strategies they used to address the risks associated with their children’s roadside behavior. Promoting pedestrian safety for children with DCD and/or ADHD necessitates collaboration among parents, schools and local authorities to implement comprehensive measures ensuring their safety. These findings contribute to understanding parental experiences and needs, providing valuable guidance for targeted interventions and policies to enhance the road safety of children with DCD and/or ADHD.

## Introduction

The ability to move around one’s community and from one location to another by any mode of transportation such as walking, cycling, driving and public transport is the definition of community mobility (Scott and Tulloch, 2021). Community mobility is an integral occupational enabler for individuals across the lifespan which supports well-being and the participation in meaningful occupations including, but not limited to, education, social participation and leisure activities (Stav, 2014; Scott and Tulloch, 2021). Independent community mobility, particularly for children, plays a crucial role in their health and physical, social and mental development (Shaw et al., 2015). As children mature, their desire for independence grows, prompting them to seek autonomy in their mobility choices. Previous research has studied the growing desire for independent mobility for children across 14 different countries (Shaw et al., 2015). By age 11 years, most children in surveyed nations could cross main roads unaccompanied, by age 12 years, a majority had the freedom to travel within walking distance alone and by age 13 years, could navigate their way home from school independently or utilize local bus services (Shaw et al., 2015). Despite the importance of community mobility, approximately 1.35 million people die every year due to preventable Road Traffic Accidents (RTA) and the World Health Organization (2018) reports that road traffic accidents are the leading cause of death for children worldwide. Thus, moving around communities can be a hazardous activity especially for groups that are vulnerable at the roadside, such as child pedestrians (Tapiro et al., 2014). In the United Kingdom, the daily average of 1 death and 10 serious injuries including children and adults has remained relatively unchanged for more than 15 years and the overall estimated cost of road traffic accidents in Great Britain is £12 billion annually (Department for Transport, 2019, 2020). Thus, safe and accessible community mobility is crucial for individuals’ well-being and meaningful engagement in activities, yet the persistent risks and high social and economic costs associated with road traffic accidents emphasizes the urgent need for effective measures to prioritize road safety.

Furthermore, previous studies have identified that children with Developmental Coordination Disorder (DCD) and/or Attention Deficit Hyperactivity Disorder (ADHD) are at additional risk of pedestrian injuries (Wilmut and Purcell, 2021; Tabibi et al., 2022). ADHD is a neurodevelopmental disorder characterized by inattention, hyperactivity and impulsivity beyond developmental norms that negatively impacts activities of daily living (Ramos-Quiroga et al., 2009). Similarly, DCD is a neurodevelopmental disorder marked by significant motor coordination impairments, adversely affecting daily activities (Kirby et al., 2013). While ADHD affects 5–7% of children globally (Abdelnour et al., 2022), the worldwide prevalence of DCD is estimated to be 5% (Blank et al., 2019). A significant co-occurrence rate exists between these two disorders, with estimates suggesting a co-occurrence of 50%, which underscores the need for investigating these disorders separately and together in relation to pedestrian safety (Goulardins et al., 2015). Navigating busy roads presents a unique challenge for children with DCD and/or ADHD. Research reveals a significantly elevated risk of pedestrian injuries for these populations compared to typically developing children (Wilmut and Purcell, 2021; Tabibi et al., 2022). This heightened vulnerability can be attributed to several key factors associated with each disorder. The characteristics associated with ADHD, including reduced attention, impulsive behaviors and hyperactivity, can have a negative influence on pedestrian performance (Wilmut and Purcell, 2020; Tabibi et al., 2022). Inattention and hyperactivity are suggested to be associated with poor timing when deciding to cross (Parr et al., 2021), while impulsive behaviors could lead to unsafe road-crossing decisions (Tabibi et al., 2022). Similarly, DCD, characterized by motor coordination impairments, presents distinct challenges for safe pedestrian behavior (Kirby et al., 2013). Deficits in spatial awareness, visual processing such as looming sensitivity, which is the ability to perceive and respond to approaching objects or vehicles, and visual-motor ability can hinder their ability to navigate complex traffic situations effectively (Purcell et al., 2012, 2017). Children with DCD may struggle to accurately judge distances and gaps between vehicles or execute coordinated movements quickly and smoothly when crossing roads leading to an increased risk of pedestrian injuries (Kirby et al., 2013; Purcell et al., 2017). For example, Purcell et al. (2011) found that poor perceptual-motor coupling in DCD can impact selecting safe temporal crossing gaps leading to inadequate crossing decisions and increased risk of injury. Additionally, children with DCD were found to have poor visual-motor abilities leading to reduced sensitivity in identifying approaching vehicles and inadequate road crossing decision-making, contributing to a potential increased vulnerability to road traffic injuries (Purcell et al., 2012, 2017). When these challenges associated with DCD combine with the inattention, impulsivity and hyperactivity observed in ADHD, the vulnerability to pedestrian accidents can further increase, potentially leading to more frequent and severe road traffic injuries (Wilmut and Purcell, 2020). In relation to ADHD, Clancy et al. (2006) found that the risk of road traffic injuries in adolescents could be attributed to inattention, although a study conducted by Stavrinos et al. (2011) found that executive dysfunction may be the primary underlying factor for the increased risk of pedestrian injuries in children with ADHD. Overall, while ongoing research continues to explore the underlying causes, there is a consensus that children with DCD and/or ADHD face an elevated risk of pedestrian road traffic accidents and injuries.

Despite the growing recognition of the risks associated with DCD and/or ADHD in relation to pedestrian safety, there is a dearth of knowledge regarding the experiences and perspectives of parents of children with these conditions in relation to pedestrian risks. Parents of these children can provide a unique perspective regarding the challenges faced in the context of road safety. Their close observation and intimate knowledge of their child’s behavior and responses to the environment, uniquely position them to offer insights into the specific challenges faced by their children as pedestrians. While behavioral studies are essential, parental perspectives provide a contextual and nuanced understanding, shedding light on the practical implications of these challenges in real-world situations. However, few studies have highlighted parents’ experiences of children with DCD and/or ADHD at the roadside. Brook and Boaz (2006) identified, through a questionnaire, that parents of adolescents, aged 16–17 years, with ADHD were more concerned about their child’s involvement in roadside accidents compared to a typically developing control group. To prevent accidents, parents suggested repeated discussions about risks, increased supervision, avoidance of dangerous play and use of medication to enhance attention and behavior (Brook and Boaz, 2006). Furthermore, Wilmut and Purcell (2020) found a relatively similar result in relation to parents of children with DCD using a quantitative parent-reported questionnaire. These parents reported that reduced attentiveness while crossing, often due to underlying perceptual difficulties, is a major concern which could manifest as a lack of confidence and increased risk-taking behavior (Wilmut and Purcell, 2020). Wilmut and Purcell (2020) further stated that the presence of ADHD characteristics in DCD was associated with further reductions in attention and increased perceived risk-taking behaviors. While these studies shed light on parents’ experiences of children with DCD and/or ADHD, there is a need for a comprehensive investigation into parental perspectives on pedestrian safety for children with DCD and/or ADHD. Therefore, the aim of this study was to explore parents’ perspectives of children with DCD and/or ADHD to gain a deeper understanding of the elevated susceptibility to pedestrian injuries among these children.

## Materials and methods

### Research aim and questions

The aim of this study was to explore the perspectives of parents of children with DCD and/or ADHD to gather their experiences of pedestrian risks. The following research questions were formulated to fulfil this aim.

- What are the perspectives of parents of children with DCD and/or ADHD in relation to their children’s ability to execute a safe road crossing?
- What, if anything, are parents of children with DCD and/or ADHD concerned about regarding their children’s pedestrian safety?
- How do parents of children with DCD and/or ADHD help to prevent or minimize their child’s involvement in pedestrian injuries?

### Reflexivity

This research adopted an interpretive, reflexive stance (Braun and Clarke, 2022). Reflexive thematic analysis is an interpretative approach to qualitative data analysis prioritizing researcher reflexivity and acknowledging the subjective nature of knowledge construction (Braun and Clarke, 2023). Therefore, the authors’ backgrounds and experiences had a profound influence on the study’s design and interpretation. To bring and own our perspectives, the first author maintained post-interview notes which played a pivotal role during the reflective analysis process in helping to understand the participants’ responses in the context of personal experiences and potential biases while serving as a valuable reference point for deeper discussion and analysis. Furthermore, ongoing discussions with the co-authors enriched insights and ensured the management of authors perspectives within the research process. Collaboration and reflection were integral aspects of our research journey, influencing various stages from design to discussion and paper editing.

### Recruitment

A purposive sampling strategy was employed to recruit parents of children with DCD and/or ADHD, this ensured participants had specific knowledge or experience relevant to the research question, enabling the collection of richer and more insightful data (Etikan et al., 2016). As such, we determined the sample composition reflecting our knowledge and understanding regarding participants’ characteristics relevant to addressing the research aim using pre-defined inclusion criteria (Thomas, 2022). This assisted in generating intensive data leading to an in-depth understanding of the experiences of children with DCD and/or ADHD as pedestrians from their parent’s perspectives.

Between January and July 2022, participants were recruited via two main avenues: social media platforms and organizations working with children with DCD or ADHD. Careful selection and display of recruitment posts on social media platforms is crucial, as inconsistent recruitment outcomes using these platforms have been reported (Topolovec-Vranic and Natarajan, 2016). Therefore, marketing headlines that trigger curiosity without compromising privacy were used to facilitate the recruitment through social media (Bender et al., 2017; Arigo et al., 2018). Furthermore, non-profit organizations, schools and institutions in the United Kingdom working with children with DCD or ADHD were utilized for recruitment. This recruitment avenue was expected to maximize access to the parents of children with DCD and/or ADHD. Ethical approval for the study was granted by the School of Healthcare Sciences Research Ethics Committee, Cardiff University. Prior to participating in the study, a pre-interview package including a participant information sheet and two screening tools were sent to potential participants.

### Measures

All participants provided written informed consent and completed the Developmental Coordination Disorder Questionnaire (DCDQ; Wilson et al., 2009) and the Swanson, Nolan, and Pelham Rating Scale (SNAP-IV; Hall et al., 2020). The DCDQ and SNAP-IV were scored according to established scoring guidelines provided by their respective authors. The DCDQ is a parent-report questionnaire designed to assess the presence of motor coordination difficulties in children (Wilson et al., 2007). It provides insights into a child’s motor ability to identify potential signs of DCD. The questionnaire includes 15 items scored on a 5-point scale, total scores range from 15 to 75 (Wilson et al., 2007). A cut-off total score of 57 or below indicates a greater possibility of motor difficulties (Wilson et al., 2007).

The SNAP-IV is a parent-report measure of ADHD and contains 26 items scored on a 4-point scale, with higher scores indicating a greater possibility of ADHD (Gau et al., 2008; Hall et al., 2020). Typically, a SNAP-IV cutoff score above 1.2 suggests an increased probability of ADHD, while a score above 1.8 is considered indicative of clinically significant ADHD (Bussing et al., 2008). These measures were utilized as part of the process to screen for the presence of DCD and/or ADHD to confirm participant eligibility for inclusion in the study. The full inclusion and exclusion criteria are summarized in Table 1 and were established to confirm that participants met the diagnostic criteria outlined in the DSM-5 for DCD and ADHD (American Psychiatric Association, 2013). Following confirmation of the presence of DCD and/or ADHD based on the pre-interview package and parental reports, interviews were scheduled at a mutually convenient time and conducted online using Microsoft Teams.

**Table 1:** Inclusion and exclusion criteria.

Inclusion criteria	Exclusion criteria
Parents of children with DCD and/or ADHD who: were aged 7–17 years had DCD and/or ADHD characteristics based on the DCDQ and SNAP-IV lived in the UK navigated the community with their children were able to communicate in English were able to provide informed consent	Parents of children who: were less than 7 years of age or greater than 17 years of age had no DCD and/or ADHD characteristics based on the DCDQ and SNAP-IV were unable to provide informed consent were unable to access to a computer and/or the internet.

### Procedure

This study utilized an online semi-structured interview approach to gather data from participants. Prior to the interview, participants were instructed to select a distraction-free environment with a reliable internet connection to ensure a smooth and uninterrupted interview process. The interview duration was typically 60 min. The interview questions were developed through an iterative process prior to conducting the interviews. In the initial stage, questions were identified by the researchers based on a review of the relevant literature pertaining to children with DCD and/or ADHD as pedestrians. Drawing on the understanding gained from in-depth reading of the topic, the questions were, then, refined, and additional questions were incorporated after discussion between the authors. Piloting was conducted to ensure that the questions were clear, comprehensible and would effectively elicit the desired information from participants. The piloting phase involved three participants, similar to the target population in terms of their roles and experiences as parents. The feedback and insights gained from the piloting were instrumental in refining and finalizing the interview questions such as rewording, enhancing their appropriateness and effectiveness for capturing the unique perspectives of parents. The final phase involved a series of iterative revisions, facilitated through discussions between authors (RF and CP), until a consensus was reached on the final set of questions that were utilized as a guide for the interviews. Examples of the interview questions are provided below.

- I would like you to walk me through your normal day while you are walking around the community with your child? What does it look like?
- Can you tell me more about your child’s behavior at the roadside and when crossing a road?
- How do you feel about your child’s performance at the roadside and when crossing a road?

The full semi-structured interview questions can be found in the Supplementary Material.

#### Coding and data analysis

To ensure trustworthiness of findings, a reflexive approach was taken to analyze the data collected from the interviews. The reflexive approach of the Thematic Analysis (TA) focuses on identifying and interpreting patterns or themes within the data through a dynamic interplay between the researcher and the research material. To achieve this, all interviews were digitally audio-recorded and transcribed verbatim to ensure accuracy and completeness of the data. The built-in audio recording and transcription features of Microsoft Teams were utilized for this purpose. The main author (RF) actively listened to the recorded interviews to ensure that the transcription was accurate correcting any inaccuracies. This approach enabled a comprehensive analysis of the data and facilitated the identification of nuanced themes.

The TA was chosen as the method of analysis, which is a widely used approach for identifying patterns and themes within qualitative data (Braun and Clarke, 2023). However, it should be noted that TA is a flexible method that can result in inconsistent and less cohesive themes if not conducted rigorously (Holloway and Todres, 2003). To mitigate this, a reflexive TA was conducted following the six phases outlined by Braun and Clarke (2023), while acknowledging the plurality of TA and recognizing that this process is recursive rather than strictly linear (Braun and Clarke, 2023). The analysis commenced with data familiarization, a first step involving an in-depth review and immersion in the data. The familiarization phase included repeated listening to the audio-recordings, reading and rereading interview transcripts to gain a comprehensive understanding, along with making initial notes and recording key ideas and emerging patterns. During the coding phase, RF systematically organized the data, assigning descriptive codes to encapsulate essential elements and meaningful information. Subsequently, RF engaged in reflective discussions with CP to ensure that RFs personal stance was consistently examined and refined in light of the emerging insights from the data. This step was conducted meticulously and recursively to ensure that the codes accurately reflected the nuances within the dataset. The subsequent third phase centered on identifying potential themes, where codes were grouped together to form overarching themes. These themes were refined by collating relevant data extracts associated with each potential theme, ensuring that they accurately represented the dataset as a whole. The creation of a thematic map followed during reviewing themes phase, allowing us to visualize the intricate connections between codes and themes. This visual representation facilitated discussions and further refinements of the themes through an iterative process, sometimes leading to extensive re-coding and re-mapping until a consensus was reached. In the fifth phase, we defined and named each theme, providing clear, descriptive explanations that enhanced our understanding of both the specificities within each theme and the broader narrative that emerged. The final stage is completion of a report which presents a coherent synthesis of the analyzed data, offering a professional and insightful representation of the themes derived from pedestrian experiences of parents of children with DCD and/or ADHD.

## Findings

A total of 14 parents of children with DCD and/or ADHD were recruited and interviewed. This included parents of 5 children with ADHD, 3 children with DCD, and 6 children with both conditions. The majority of the participants children were male, accounting for 71.4% of the sample, while 28.6% were female. Participants’ children demographics are summarized in Table 2. This demographic information presented in Table 2 provides a more detailed view of the children’s age range and specific diagnosis.

**Table 2:** Demographics of the participants children.

Primary Condition	Gender of the child		Children’s age range	Total number of participants
	Male	Female		
ADHD	3	2	7–13	5
DCD	1	2	10–17	3
DCD and ADHD	6	0	7–16	6
Total	10	4	7–17	14

Table 3 provides an overview of the participants, including pseudonyms used for their names and children’s characteristics.

**Table 3:** Parents’ information (pseudonyms used for names).

ADHD		DCD		ADHD + DCD	
Parents’ names	Children info	Parents’ names	Children info	Parents’ names	Children info
1- Alex: 2- Ava: 3- Olivia: 4- Sophia: 5- Emily:	daughter 9 years old son 12 years old daughter 9 years old son 13 years old son 7 years old	1- Isabella: 2- Mia: 3- Lily:	daughter 17 years old son10 years old daughter 16 years old	1- Maryam: 2- Charlotte: 3- Harper: 4- Amelia: 5- Evelyn: 6- Harry:	son 10 years old son 16 years old son 7 years old son 7 years old son 13 years old son 8 years old

The collected data on parents’ perspectives of children with DCD and/or ADHD at the roadside were analyzed, and three distinct themes were developed. The first theme explored parents’ observations of their children’s roadside behavior and road-crossing performance, revealing unique challenges related to these conditions. The second theme examined parental perceptions, unveiling their concerns and emotions about their children’s pedestrian safety. The third theme highlighted parents’ resourcefulness in crafting survival strategies to safeguard their children.

**Figure 1:**
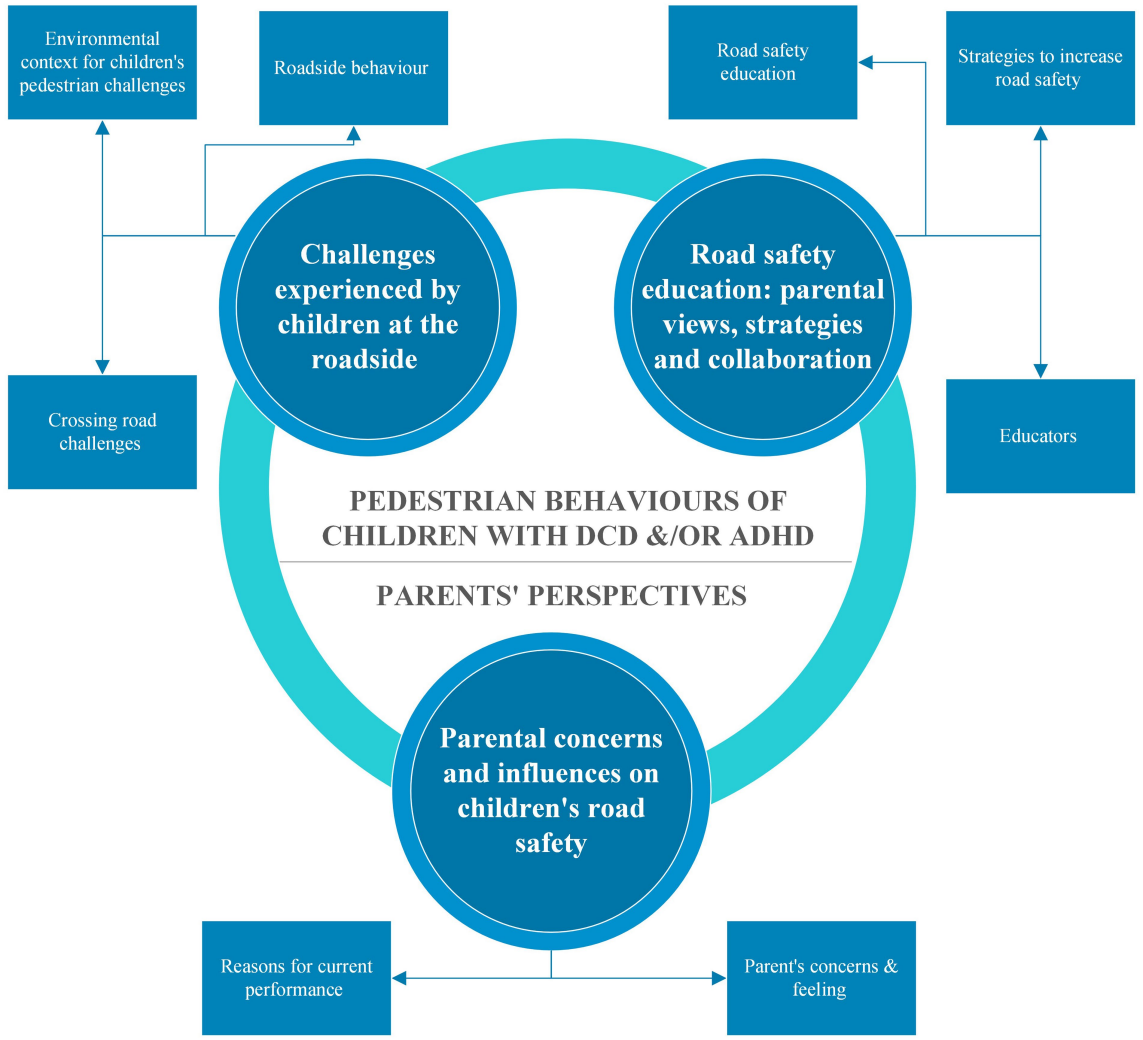
Summary of the themes and sub-themes.

These themes shed light on parents’ perspectives of children with DCD and/or ADHD concerning pedestrian roadside safety. A summary of the generated themes and sub-themes are presented in Figure 1. These will now be discussed.

### Theme 1: challenges experienced by children at the roadside

This theme highlights descriptions parents of children with DCD and/or ADHD gave in terms of their children’s roadside behavior and road crossing performance. Although many participants experienced similar pedestrian environments, parents from each group reported different pedestrian behaviors which related to whether their child had DCD and/or ADHD.

### Environmental context for children’s pedestrian challenges

To provide context for a deeper understanding of their children’s pedestrian challenges, we begin by exploring the environmental context in which these challenges unfold. Among the 14 participants in the study, 10 resided in urban areas, while the remaining 4 lived in rural or village settings. However, participants from rural areas lived in close proximity to a town and were exposed to similar transport infrastructure. One participant, Sophia from the ADHD group, described living in a:

*“…village or close to countryside but there are transport infrastructure busses, roundabout, signalized crossing, zebra crossing, and alleyway” (13-years old)*.

All participants agreed that zebra crossings, signalized crossings and human controlled crossings are safer crossing sites when compared to midblock crossing sites.

*“For signalized crossing, he likes to press the button and waits” (Emily, 7-years old, from the ADHD)*.

*“those [referring to Zebra and signalized crossing], she’s okay because all you have to do is wait for the cars to stop, do not you? And when you can clearly see that they have stopped, then you can go. So, the decision …. She’s not having to judge” (Isabella, 17-years old, from the DCD group).*

*“He will wait by the lollipop lady and he knows because he’s learning that rule” (Maryam, 10-years old, from the co-occurring group).*

However, parents of children with ADHD expressed concern about their children’s safety when crossing driveways due to previous near-miss incidents.

*“The most important is the driveways as Emma runs across all those driveways.” (Olivia, 9-years old, from the ADHD group).*

Parents in the ADHD group also reported that their children exhibit less dangerous behaviors when using zebra and signalized crossings as long as they are not distracted. However, parents reported that children with ADHD tend to exhibit risky behavior, such as standing at the edge of the pavement.

*“He does stop, but what he does do is he stands at the very edge of kerb. So, he will really push the limit and be like, I am here.” (Emily, 7-years old, from the ADHD group).*

Conversely, parents reported that children with DCD showed a greater inclination to wait for others to cross with them at these crossing sites. Parents reported that they followed their parents, groups of pedestrians and the instructions of road crossing patrols.

*“Because she’s frightened of the traffic, she’ll just follow other people [other pedestrians]” (Lily, 16-years old, from the DCD group).*

According to parents, children with co-occurring DCD and ADHD also relied on others when using zebra crossings, signalized crossings and human controlled crossings, unless they were distracted by something on the other side of the road.

*“He will wait by the lollipop lady and he knows because he’s learning that rule, that he sits and waits there for me. So, he will never cross without me” (Maryam, 10-years old, from the co-occurring group).*

In summary, parents highlighted similar pedestrian infrastructures in both urban and rural areas. However, they articulated a range of challenges experienced by their children with DCD and/or ADHD in relation to these infrastructures. While zebra crossings and signalized crossings were considered safer, concerns about driveways were common among parents of children with ADHD, highlighting potential attentional issues characteristic of ADHD. Children with ADHD tended to exhibit risky behaviors near pavements possibly due to difficulties associated with hyperactivity, whereas those with DCD showed a greater inclination to wait for other pedestrians or pedestrian signals leaving the crossing decision to other people or road architecture. This could indicate that children with DCD have less confidence in their perceptual and motor abilities. Children with co-occurring DCD and ADHD showed a combination of these behaviors such as relying on others for crossing and distracting easily.

### Roadside behavior

Common roadside behaviors reported by parents across the three groups were difficulties when multitasking, such as talking and walking, which could exacerbate the problem and negatively affect their roadside safety. For example:

*“When he is absorbed in what he is doing,* e.g.*, talking to his brother, I need to grab his attention first by tapping his shoulder or the back of his neck, or I’ll try and get near him and say, like, hey, John, just to try and cut through what’s going on in his brain.” (Emily, 7-years old, from the ADHD group).*

*“It’s that concentration she cannot seem to multitask. She cannot talk and walk at the same time because it just takes her concentration away from what she’s doing.” (Lily, 16-years old, from the DCD group).*

Furthermore, parents of children with ADHD described their children as very active, energetic and tending to run around, jump and engage in non-stop talking, making it difficult to focus on road safety. As a result, parents observed their children running or walking without noticing the edge of the kerb, leading to walking or running on the road instead.

*“But she’s that energetic and she’s that bouncy. She will run ahead and go straight across the driveway without even thinking that the pavements changed” (Olivia, 9-years old, from the ADHD group).*

*“They [John and his brothers] just like wander off the pavement into the road and start walking in the road instead of on the pavement. Especially John likes to walk right on the edge of the kerb.” (Emily, 7-years old, from the ADHD group).*

On the other hand, parents of children with DCD reported a lack of spatial awareness, which often resulted in them bumping into people and things. As parents report, they tend to rely on someone walking beside or in front of them. As a result, parents suggested that they lacked confidence in their children’s ability for independent mobility and decision-making at the roadside. Examples from parents when discussing walking on the pavement include:

*“She’s always covered in bruises, where she’s constantly bumping into things.” (Lily, 16-years old, from the DCD group).*

*“She always tends to stay by the side of someone. She’s never one to take the lead. If this only one person can fit through, she tends to stay back and follow.” (Isabella, 17-years old, from the DCD group).*

Parents of children with co-occurring DCD and ADHD reported a combination of these behavioral characteristics. While they were reported to have similar characteristics to the DCD group, including relying on others for decision making and exhibiting poor spatial awareness, leading to children bumping into people and things, those with co-occurring DCD and ADHD were described as often running, jumping and climbing. They were also observed to be unaware of the edge of the kerb, which increased their likelihood of walking on the road.

*“So, I’m always reminding him to walk near the wall, not the edge of the road. All the time bumping into people. So, he’s just got no concept of where his body is” (Maryam, 10-years old, from the co-occurring group).*

*“Because of the big thing with Malcom is his spatial awareness. He does not realize where his body is in the space. He is just sort of floating about and he would just not realize that the pavement had ended, and the road had start.” (Amelia, 7-years old, from the co-occurring group).*

This sub-theme highlighted distinct challenges faced by parents of children with DCD, ADHD and co-occurring DCD and ADHD regarding road behavior. Parents across the three groups commonly reported challenges with multitasking during roadside activities, which could worsen the situation and pose risks to roadside safety. Parents of children with ADHD noted their high activity levels, making them prone to running or walking on roads without noticing hazards. Conversely, parents of children with DCD reported a lack of spatial awareness, leading to frequent collisions with objects/people. Those with co-occurring DCD and ADHD were reported by parents to display a combination of both behaviors, poor spatial awareness and high activity levels, emphasizing the complexity of pedestrian safety for these children.

#### Crossing road challenges

The behavior of children with DCD and/or ADHD when crossing roads was also reported by parents. Parents of children with ADHD discussed how their children tend to run or walk straight across the road without looking at oncoming vehicles or checking both ways. Parents believed that their attention is often directed toward their intended destination only, resulting in a disregard for the environmental cues and hazards in the immediate surrounding environment. This phenomenon was frequently referred to by parents as “tunnel vision.”

*“Not able to pay attention around about his surroundings, so he will literally just walk straight across the road without looking, he does not look” (Ava, 12-years old, from the ADHD group).*

*“When I went to pick him up from school, he ran into the road not looking.” (Sophia, 13-years old, from the ADHD).*

*“She’s just she tends to look at the point she wants to get to and it’s almost like she gets tunnel vision. Nothing else is there. She just needs to get from where she is to that thing over there” (Olivia, 9-years old, from the ADHD group).*

All parents of children with DCD, on the other hand, noticed their children move their head right and left as a visual scan before crossing but have difficulty in interpreting the visual cues to make an appropriate decision.

*“She would turn her head like this. But she wasn’t actually looking at the cars and making a judgment. She was doing the movement. And she would stand there, and she would do the head movement only.” (Isabella, 17-years old, from the DCD group).*

The parents further explained that they either rely on other pedestrians to make the decision or they make a random decision to cross the road, thereby increasing their vulnerability to accidents.

*“She has not got the confidence and because she’s frightened of the traffic, she’ll just follow other people [other pedestrians], which sometimes is not a good thing because if they run out in front of something, she’s trailing behind” (Lily, 16-years old, from the DCD group).*

Parents reported observing their children either running across or looking down during road crossing. Parents suspect that children lack confidence in their decision-making ability and we assume that may contribute to the variation in their crossing styles. Moreover, they stated children with DCD lack the ability to judge the speed of an approaching vehicle and cannot determine whether vehicles are far enough away to safely cross the road.

*“She walks head down, she’s like, I made my decision, and my head is down, and I am going. She does not run but she does walk very fast” (Isabella, 17-years old, from the DCD group).*

*“She cannot make that judgment. She cannot tell if that car is far enough away.” (Lily, 16-years old, from the DCD group).*

According to parents, children with both DCD and ADHD demonstrated relatively similar behaviors to the ADHD group and DCD group. Children may look both ways before crossing but face difficulties in processing the visual cues to make an appropriate decision.

*“So, he was standing at the edge of the road, and he would turn his head. But he would not necessarily spot that the car was coming” (Maryam, 10-years old, from the co-occurring group).*

Similar to the DCD group, parents of children with DCD and ADHD observe safe road crossing when among a group of pedestrians and tend to walk behind other people. Furthermore, children with DCD and ADHD were described as experiencing difficulties in judging the speed of approaching vehicles relative to their own speed, potentially leading to dangerous situations.

*“I do not think he can judge how fast he’s going. You know, if there’s a car coming, if there’s a car at the end of the road, he would not know that car was far enough away that you could cross.” (Maryam, 10-years old, from the co-occurring group).*

Parents also observed their children stopping in the middle of the road or expecting the car to stop for them, like at a zebra and signalized crossing.

*“He does not really know how to react to traffic, so I’m trying to give an example of something that happened. We were crossing quite a busy road and as we were crossing the road, he stopped right in the middle of the road because there was a bus approaching. But it was a red light so the bus would have stopped but he stopped dead in the middle of the road” (Amelia, 7-years old, from the co-occurring group).*

Parents of children with DCD and ADHD reported that their children might engage in impulsive behavior when crossing roads. For instance, they may exhibit a tendency to run across the road if they perceive something of interest on the other side or if they recognize someone they know.

“*He might run across the road if he saw somebody he knew on the other side, or a dog that he wanted to speak to, he would not think it was a road.” (Maryam, 10-years old, from the co-occurring group).*

Overall, it is evident that visual-motor/attention challenges play a substantial role in the pedestrian safety of all children in the study. Whether due to issues related to attention or as described “tunnel vision” (in the case of ADHD), difficulties in processing visual cues (in the case of DCD), or a combination of these factors (in the case of co-occurring ADHD and DCD). These challenges underscore the importance of addressing visual perception and attention in enhancing road safety for these children.

### Theme 2: parental concerns and influences on children’s road safety

Parental perceptions are critical aspects in understanding parents’ perspectives regarding their children’s road safety behavior and performance. This theme explores parents’ concerns and feelings about their children’s pedestrian safety and possible underlying causes of roadside performance through two subthemes.

#### Parent’s concerns and feelings

The first subtheme revealed that parents of children with ADHD experience apprehension regarding their child’s independent travel abilities, even when utilizing public transport from remote locations, such as busses.

*“We’ve just been quite nervous about him doing that on his own and organizing himself to get on a bus that’s 10 miles away to come home. Do not feel quite yet, he’s ready to do that. So, I suppose we supervise him a lot and he does not really go anywhere on his own without us.” (Sophia, 13-years old, from the ADHD).*

These parents monitor their children closely and restrict them from going out unsupervised. They also frequently hold their child’s hand, fearing potential traffic accidents.

*“I probably held his hand a lot because I was very worried about him running off onto the road” (Sophia, 13-years old, from the ADHD).*

Furthermore, the situation may be further complicated by the fact that the parent may be a single parent with the child and their siblings, making it even more challenging.

*“I would assume that it is usually me with my three kids. So, it’s not only me and John. So yeah, it it’s kind of hard.” (Emily, 7-years old, from the ADHD group).*

Parents of children with DCD noted that road crossing may not be a priority initially, due to other pressing developmental issues, but as their children enter adolescence, the significance of safe road crossing becomes increasingly apparent. These Parents expressed concern about their children’s ability to navigate roads safely.

*“When she was very small, it wasn’t something we thought about so much because of the range of problems that Claire had, it was not a top priority. We had other more pressing issues like skills, milestones that were late, that were more important than crossing the road. It really started to be an issue. I think when she got to be a teenager. So, by the time she was in secondary school, 12 could not cross the road and it wasn’t even close to being able. When we attempted to teach her to cross the road. She wasn’t even close to being able to do it safely and to make safe decisions” (Isabella, 17-years old, from the DCD group).*

*“What happens as the children get older is that their independence is very restricted by this. Basically, a child that cannot cross the road cannot leave the house on their own. And you suddenly find that you have this child, who is 16, who is 5 feet tall who still needs their mum to take them.” (Lily, 16-years old, from the DCD group).*

These concerns can limit their children’s independence leading to increased parental supervision when they wish to become independent. Moreover, parents stated driving children to school can hinder their development when they want their children to gain more road safety experience. Therefore, parental’ concerns regarding their children’s road safety are difficult when balancing children’s independence with road safety. Based on insights shared by parents, effective strategies to teach their children how to cross roads safely while still allowing them to develop the independence they require for community mobility can facilitate this goal.

*“I do not think it’s helped because, we have had to take her back and forth to school every single day because of the distance of the school from where we live to where the school is and like I said, there’s no way she could have walked safely back and forth. It’s way too far and the roads are too busy, and I do not think it’s helped her, and this is why I want to really encourage her to start going out with her friends now. So, because the more experience she’s got on the road, the better it’s going to be for her. She cannot live in a bubble with her parents walking behind her for the rest of her life.” (Lily, 16-years old, from the DCD group).*

Likewise, parents of children with co-occurring DCD and ADHD expressed concern and fear for their children’s road safety. These concerns demonstrate the complexities and challenges of fostering independence in children while ensuring their safety. Parents recognized that their children’s safety depends not only on their own actions but also on the actions of others, such as drivers and pedestrians.

*“It’s my biggest fear that Malcolm is going to get run over because it’s so likely to happen. I can see it happening based on how I see him every single day near roads. As he gets older, and he becomes more independent, and he starts wanting to do more things independently, my fear grows.” (Amelia, 7-years old, from the co-occurring group).*

Additionally, parents were aware of the impact of their child’s mood and fatigue levels on their judgment, making it essential to consider their emotional and mental well-being in addition to their physical safety.

*“So generally, I’m quite nervous and scared, but some days he’s good, really good and really receptive and really responsive. And then the other days are just because he gets fatigued in the afternoon because of how on-the-go it is in the afternoon, it’s like a bit of a clouds formed his judgment and his mind. So, it depends on the time of day, and it depends on his mood.” (Amelia, 7-years old, from the co-occurring group).*

Overall, this sub-theme highlighted parents’ concerns of children with DCD, ADHD and co-occurring DCD and ADHD in ensuring their child’s safety during road crossing. Parents of children with ADHD expressed concern regarding their child’s independent travel abilities, leading to increased parental supervision and a possible decrease in the opportunity for children to learn safe road crossing behaviors. Parents of children with DCD initially prioritized other developmental issues over road crossing but later express concerns about their child’s ability to navigate roads safely. Parents of children with co-occurring DCD and ADHD also described their fear for their children’s road safety, emphasizing the complexities of fostering independence while ensuring safety. Thus, all parents faced the demanding challenge of finding the balance between fostering their children’s independence while prioritizing their pedestrian safety. The parents’ expressions of fear and concern illustrate the importance of recognizing the complexities of the road crossing task and the need for effective strategies to teach children how to cross roads safely as they develop independence.

#### Reasons for current performance

Parents of children with ADHD perceive their children’s poor performance as pedestrians to be linked to ADHD characteristics, which is affected by their mood and temperament. Specifically, parents reported that during episodes of bad mood or inability to self-regulate, their children’s impulsiveness and inattention of their surroundings may lead to unsafe crossing behaviors, such as darting across roads without checking for oncoming traffic.

*“Worse during the bad temperament, more like oppositional defiant disorder, leading to not stop and shoot across roads and I think he did not hear anything around.” (Ava, 12-years old, from the ADHD group).*

However, during periods of good mood, these parents reported that their child’s road safety behavior could be adequate, highlighting the critical role of mood fluctuations in performance. Despite these challenges, parents expressed optimism about their children’s ability to develop strategies as they grow older leading to improved pedestrian performance.

*“If she’s in a good frame of mind, she’s like a professor of it. She will tell you exactly how you should cross the road. But if she’s in a bit of a bad mood or whatever, it’s like fight or flight response, and the matter of fact is that she can tell me perfectly she will just go.” (Olivia, 9-years old, from the ADHD group).*

Similarly, all parents of children with DCD believed that their child’s poor pedestrian performance is related to their inability to judge distance and speeds accurately. They also reported that their children may struggle with spatial awareness, which can make it difficult to navigate around obstacles and people on the pavement. Additionally, fatigue, lack of confidence and forgetfulness can further impact their children’s judgment and spatial awareness.

*“She [referring to Isebella’s daughter] cannot always tell where other people are properly. So, walking into people is a real problem. So, she constantly has this fear that people are gonna walk into her because she cannot tell where she is. So, she does not know where they are.” (Isabella, 17-years old, from the DCD group).*

*“My feeling is that she cannot judge the speed of the car. So, you know if you or I look, you can tell and you learn through experience how fast the car is going. And is that car far enough, and have I got time to cross?” (Isabella, 17-years old, from the DCD group).*

For parents of children with both DCD and ADHD, the DCD and ADHD are perceived to be contributing factors to their children’s poor performance as pedestrians. They expressed concern about their child’s lack of focus and impulse control, which can lead to unsafe behavior on the road. Moreover, parents acknowledged that their child’s spatial awareness, concentration, attention difficulties and motor skills may affect their ability to judge distances and navigate their environment safely.

*“If he’s tired, if he’s worried, you know? So, if he’s anxious about something, then he’s more likely to be dysregulated on edge and more bouncy, and all over the place, as we say” (Harry, 8-years old, from the co-occurring group).*

*“I think it’s both [referring to ADHD and DCD]. So, the impulse bit is obviously ADHD, but because he has no idea of what his body is doing, you know he cannot stay upright. He does not know where he is in space, which is more of the DCD I think…When he’s focused, he can cross the road safely. But you never know which day he’s going to be focused, or you know which minute he’ll be focused, and which minute he will not.” (Maryam, 10-years old, from the co-occurring group).*

Despite these challenges, some parents remained hopeful that with time and support, their child can improve their pedestrian performance. However, they currently would not feel comfortable allowing their child to walk to school independently or with friends, as they believe it would be unsafe.

*“So next year, when he goes to secondary school, I will not let him walk to school on his own or even with a group of friends because I would not put them in that position where they have to keep him safe.” (Maryam, 10-years old, from the co-occurring group).*

In general, parents of children with ADHD link poor pedestrian performance to ADHD traits such as impulsivity and inattention which is affected by mood fluctuations. For those with DCD, spatial awareness, motor skills and fatigue pose challenges for appropriate pedestrian behaviors. Children with both conditions face a combination of limitations related to concentration, attention difficulties, motor skills and spatial awareness, leading to significant parental concerns about their road safety.

### Theme 3: road safety education: parental views, strategies and collaboration

This theme explores parents’ perspectives of current road safety education and the various strategies they developed themselves to ensure their children’s safety. Despite concerns, parents have developed invaluable pedestrian safety strategies that can be adopted to enhance the practical aspects of road safety education.

#### Road safety education

Parents of children with DCD and/or ADHD discussed their children’s road safety education and also suggested some elements to facilitate the effectiveness of the education. Parents of children with ADHD believed that while their child received some road safety education, it may not be sufficient. They suggested that stories, which provide an emotional connection, may have a greater impact on their child than generic videos.

*“I know they had that Bobby Colleran [campaign] that Slow Down for Bobby [Bobby Colleran is a local road safety campaign in the UK aimed at promoting safe travel to schools]. The family go to her school, and they were there a couple of weeks ago, going through everything again and sharing the books and we have got the books at home as well. We’ve read them… So, she’s [Olivia is] quite compassionate, so that will make her think more than just watching a video of someone that she does not know.” (Olivia, 9-years old, from the ADHD group).*

*“You know the old adverts that used to be on the television about if you did not buckle in… we had to show her things like that to make her understand the implications of those choices. From that point onwards, she wore a seat belt. No question. She gets straight in and buckles up.” (Olivia, 9-years old, from the ADHD group).*

Moreover, parents stressed the importance of teaching road safety in a way that their child can understand, with step-by-step instructions and minimal distractions. They reported that one-to-one or small group sessions can be most effective.

*“Instructions need to be step by step, otherwise, his brain gets overloaded.” (Emily, 7-years old, from the ADHD group).*

Parents of children with DCD stated that they found ways to adapt to their children’s needs. They were involved in the school’s Kerbcraft program to reinforce road safety practices and took the initiative to teach their child about road safety during outings.

*“Well, in school they do the same thing. They do Kerbcraft once from five or 6 years of age where they take them out in the community, and they cross busy roads.” (Lily, 16-years old, from the DCD group).*

They also mentioned a transition training program preparing their child for the transition from primary to secondary school, where road safety awareness is essential. Additionally, they emphasized the active role of parents in teaching their children road safety.

*“They do a transition at the last year. So, when they go from year six to year seven and the road safety officer will go into the primary schools and speak to them and tell them, think how you are going to get to your new school when you start in September, plan your route, look for the safest route, do not look for the shortest route, it’s gotta be the safest route. So, she did that as well.” (Lily, 16-years old, from the DCD group).*

While parents of children with both DCD and ADHD saw road safety education as crucial, their experiences revealed significant challenges in transferring theoretical knowledge into practical implementation. These parents, for example, reported that their children had received some road safety education at school and scout club. However, they agreed that their children might not put what they had learned into practice, particularly if they were not familiar with the roads.

*“Theoretically, it does not work. For it to be slightly muscle memory, you need to do things physically. And that means practicing it, getting in the habit of doing it, and seeing it in practice.” (Amelia, 7-years old, from the co-occurring group).*

They further believe that simulation, one to one training and a training program with movement activities and visual materials would be more effective.

*“Somehow exposing him to dangers in a safe way. Like through simulation, might help him understand and remember road safety lessons better. You can do visual social stories with him, but he does not relate to that if it is presented on a page, I think you know if you put him through some kind of road safety simulation, that would work because he’d remember it and he’d be in it.” (Maryam, 10-years old, from the co-occurring group).*

*“I believe that learning should come from an angle where it’s kind of like a visual, auditory, reading, and kinesthetic experience. So, it would involve a lot of movement, a lot of walking around, a lot of like activities, things that are visual, things that give context to the situation. You know, like when they do, first aid, you have gotta practice doing the CPR and stuff like that” (Maryam, 10-years old, from the co-occurring group).*

*“You’re talking one teacher to 30-odd children, so Malcolm does not concentrate very well in school but he benefits greatly from one-on-one support.” (Amelia, 7-years old, from the co-occurring group).*

Some of them proposed that such training should be continuous, daily and revisited every few weeks or months with catchy campaigns and ads promoting road safety education. They also emphasized the importance of preparing their children for adulthood and for independent pedestrian mobility.

*“Missed days with Malcom, it becomes out of his routine and then you just gotta start again. So doing things daily is really important. In school, it might be different, though. Say, if they were doing lessons on road safety in school, I think weekly would be fine if they got into a habit of it.” (Amelia, 7-years old, from the co-occurring group).*

In discussing road safety education, parents of children with DCD and/or ADHD highlighted various challenges specific to their respective group of children. Parents of children with ADHD emphasized the need for more impactful educational materials, such as emotionally engaging stories, to supplement existing programs. They also stressed the importance of providing step-by-step instructions and minimizing distractions during training sessions. Parents of children with DCD described their efforts to adapt to their child’s needs, including participation in school-based programs like Kerbcraft and transition training. They emphasized the significance of practical training and one-on-one support, as well as the need for continuous and frequent reinforcement of road safety concepts. Parents of children with co-occurring DCD and ADHD expressed similar viewpoints, underscoring the importance of personalized training approaches that incorporate visual and kinaesthetic elements. They also advocated for increased frequency and scope of road safety campaigns, along with real-life stories to highlight the risks associated with road traffic accidents. Overall, while parents across all groups recognized the importance of road safety education, they identified specific challenges and recommended tailored approaches to address the unique needs of their children.

#### Strategies to increase road safety

Parents of children with DCD and/or ADHD developed various strategies to mitigate the risk of pedestrian injuries. Table 4 contains a summary of the strategies used by parents. Parents of children with ADHD expressed the need for constant forward planning to reduce the risk of pedestrian injuries. They select quieter roads when possible and minimize the number of roads their child needs to cross. Some parents opted to drive their child to school and drop them off on quieter roads due to their child’s behavioral characteristics.

**Table 4:** Parents’ strategies.

Group	Strategies mentioned by parents
ADHD	Constant forward planning Selecting quieter roads Driving to school Verbal cues and prompts Assigning responsibility Use of disability blue badges Crossing with peers Walking in the middle of the group
DCD	Repetition or repeated practice Familiarizing with environment and roads Crossing with peers Focus teaching on specific roads Avoiding traffic
ADHD + DCD	Verbal cues and prompts Hand gestures Physical guidance Repetition or repeated practice Familiarizing with environment and roads

*“It’s just having to constantly forward plan, even if it’s just like a walk before bedtime, it’s constantly forward planning because you can just never plan what she’s gonna do.” (Olivia, 9-years old, from the ADHD group).*

*“If there’s a certain way, we’ll walk, and we’ll try and do it to the way where there’s less roads to cross.” (Alex, 9-years old, from the ADHD group).*

To draw their child’s attention to the road, parents often use verbal cues, such as talking through the situation and shouting ahead. Additionally, assigning responsibility to their child, such as asking the child to tell them when it is safe to cross the road, was reported to help children with ADHD to pay more attention. To increase safety, some parents use disability blue badges to park closer to their destinations. Crossing with peers and walking in the middle of the group were reported as tactics they use.

*“you’d have to shout ahead. So, lots of talking it through, drawing his attention to look at the road.” (Sophia, 13-years old, from the ADHD).*

*“I need to grab his attention first by tapping his shoulder or the back of his neck, or I’ll try and get near him and say, like, hey, John, just to try and cut through what’s going on in his brain” (Emily, 7-years old, from the ADHD group).*

*“So, like if I ask him say like, you are gonna tell us when it’s OK to cross the road, he will give more attention to the task” (Emily, 7-years old, from the ADHD group).*

Parents of children with DCD emphasized the importance of repetition and familiarity in mitigating the risk of pedestrian injuries. They noted that their child would become overcautious in unfamiliar places and wait until there was no traffic before crossing the road. To address this, they identified the roads their child would need to cross and familiarized them with these roads, starting at a quiet time of day. One parent described how they repeatedly crossed the same main road with their child for about two weeks until the child was comfortable making the decision to cross safely.

*“We went down to the main road and we just crossed it again and again and again. And all we did every day for like an hour a day, for about 2 weeks, was to just go to that road, cross to one side and then cross back again and then cross back again. But it was forcing her to make the decision. So, it was very time consuming.” (Isabella, 17-years old, from the DCD group).*

Due to their child’s need for repeated learning, the parents emphasized the importance of repeating the practice several times to ensure their child was familiar with the route. Another parent mentioned that they would walk the route with their child several times to ensure they were comfortable and familiar with it before allowing their child to volunteer in that area.

*“With her DCD, she needs to have repeated learning. So, you could not just do it once and then think she’s OK. It needs to be repeated. So, four or five times because she’s… for example, she’s volunteering for play scheme to look after younger… So, we are gonna walk it about three or four times with her to make sure that she’s familiar with the route.” (Lily, 16-years old, from the DCD group).*

Isabella further suggested implementing environmental changes such as adding more zebra crossings on main roads, particularly at roundabouts, to increase safety. She also suggested the use of indicators on the kerb to identify the safest place to cross. For example, these indicators could be a distinct marking on the pavement edge, clearly indicating the safest area for pedestrians to cross.

*“I think the other thing that’s helpful with zebra crossings and Pelican crossings is because it’s like a set piece wherever you go… I think we need more Zebra crossings on main roads.” (Isabella, 17-years old, from the DCD group).*

However, parents noted that transferring skills from one road to another is challenging for their children. To address this issue, they suggested changing the teaching mindset from general life skills to specific roads that the child needs to cross.

*“So that for every road that she’s gonna need to cross, she has to work out a specific sort of skill set for that particular road.” (Isabella, 17-years old, from the DCD group).*

Finally, parents reported avoiding traffic whenever possible, for example, by driving to school earlier to park in a safer location.

*“After the near misses. What I started doing was I started driving to the school 20 min earlier so that I could make sure I could park somewhere where she would not have to cross the car park to get to the car.” (Isabella, 17-years old, from the DCD group).*

*“She’ll only go at certain times of day, so she would not go when it was busy. So, sort of, you know, 4:30 she would not go because the road is too busy. So, she’s picking times when it’s not busy.” (Isabella, 17-years old, from the DCD group).*

Parents of children with both DCD and ADHD also discussed strategies to minimize the risk of pedestrian injuries. Verbal prompts were considered useful, as they help create awareness for the child, but constant reminders were necessary, especially when crossing the road.

*“There is some awareness, but I always have to remind him to look both ways, because there’s always that possibility that a car may not stop still. So, yeah, there’s a lot of prompting… so I’ll give him a heads up if we are going to go left or we are going to cross. So, sort of hand gestures.” (Evelyn, 13-years old, from the co-occurring group).*

Hand gestures were used to indicate the direction of movement and sometimes physical guidance was needed to help the child stay on track, especially when navigating unfamiliar environments. Repetition was also found effective, with one parent (Evelyn) noting progress over the past year by practicing crossing two specific roads to school every morning. Familiarity with the environment can also play a role, as the child may feel more confident and aware in familiar surroundings.

*“Yeah, I’ve noticed progress definitely, especially over the last year in the two roads that we crossed to get to school with practice every single morning crossing those roads and when I’m with him, I’m telling him what to do and I’m watching him and trusting him to cross the roads safely. But it’s gonna be a long time before we get to in being able to do anything like that on a busier road.” (Evelyn, 13-years old, from the co-occurring group).*

*“It depends where he is. It depends whether he’s familiar with that environment.” (Charlotte, 16-years old, from the co-occurring group).*

Overall, parents employed a range of strategies to ensure their child’s pedestrian safety according to their needs. Children with ADHD thrive with forward planning, verbal prompts and attention management techniques, while those with DCD benefit from repetitive practice, familiar routines and environmental modifications. For the unique challenges posed by co-occurring DCD and ADHD, parents blend these approaches, adapting to unfamiliar situations, noting incremental progress.

#### Educators

The parents of children with DCD and/or ADHD discussed the responsibility of delivering road safety education for their children. Parents of children with ADHD believed that involving professionals such as the police and transport companies were crucial, in particular courses for bus drivers would be beneficial since busses are often used to transport children to and from school. Various delivery options were discussed, but parents believed that they ultimately had the most frequent opportunities to put education into practice and walk with their children every day, while teachers and Scout leaders could reinforce the message.

*“Parents, teacher and Beaver’s leader but the parents have the responsibility more than any other as they have more opportunity to put everything in practice and they walk everyday. Teacher and others should re-enforce.” (Emily, 7-years old, from the ADHD group).*

When it comes to children with DCD, the parents felt that the responsibility of delivering road safety education fell on them, as they knew their children best and could adapt to their needs accordingly. However, they also suggested that local authorities could play a role in delivering extra lessons in schools to supplement what parents were teaching at home, ensuring that children with DCD were equipped with the necessary road safety skills.

*“This is our job, you know. You are the parent and yes, it takes longer with these children. But you know that’s called being the parent of a dyspraxic child” (Isabella, 17-years old, from the DCD group)*.

*“And with active travel, it’s all very well put these roots in place, but if people are not learning their children to use them because the children got disabilities and they cannot use them, I think local authorities should help step in. I know the primary responsibility lies with parents at the end of the day, it is their children and they should make sure they are safe. But I think if the local authority can help by delivering extra lessons in school, it might be beneficial.” (Lily, 16-years old, from the DCD group).*

For children with ADHD and DCD, some parents believed that external professionals like the police would be more effective, while others suggested involving both parents and teachers. They also agreed that road safety education should be accessible to parents at home and delivered in schools by teachers, with the suggestion that teachers could incorporate it into the curriculum. While some parents believed that an external professional was needed, others felt a teacher with proper guidance documents could deliver a program effectively. Therefore, the parents felt that schools should take the lead in delivering road safety education, with parents and teachers working together to ensure their children’s safety on the road.

*“I think he probably responds better to external people. You know, if the police came and did it, he listens. You know the problem with parents doing things like that is you only have so much time in your day to do the things you need to do including therapy as well.” (Maryam, 10-years old, from the co-occurring group).*

*“I think what would be really helpful if it’s something that parents could access themselves, but also to be delivered in schools.” (Harper, 7-years old from the co-occurring group).*

*“By default, they are probably say at schools, but I think if you can get into computer games, something where it’s leisurely and does not feel like it’s forced upon them. Because sometimes if you put it through, say, it’s just for schools, it just schools teach it. It does not necessarily go in because it feels like you are forcing it in for information upon me instead of me understanding that it’s valuable to me in life. So, it’s getting that balance of doing it in a way that feels like it’s fun.” (Evelyn, 13-years old, from the co-occurring group).*

Parents of children with DCD and/or ADHD discussed the responsibility of delivering road safety education for their children. Parents of children with ADHD felt that it was important to involve professionals like the police and transport companies, while also acknowledging their own role in daily practice. Parents of children with DCD felt primarily responsible but suggested local authorities supplement education. For children with co-occurring DCD and ADHD, parents had mixed views on involving professionals versus teachers, but agreed on the importance of accessible education delivered at home and in schools. Overall, ensuring road safety for children with DCD and/or ADHD was seen as a responsibility shared by parents, schools and local authorities. While parents saw themselves as playing a critical role in adapting education to their children’s needs and reinforcing the message, schools can provide accessible education to supplement what parents teach at home. Additionally, local authorities can offer extra lessons and support to ensure children have the necessary skills to navigate the roads safely. By working together, parents, schools and local authorities can ensure the safety of all children on the roads.

## Discussion

The aim of this study was to explore the perspective of parents of children with DCD and/or ADHD with the goal of gaining a better understanding of the pedestrian risks faced by their children. Semi-structured interviews were conducted with parents and three main themes were generated, each aligning with a specific objective. In the first theme, parents’ perspectives of the challenges faced by children at the roadside, addressed the objective of exploring the unique perspectives of parents of children with DCD and/or ADHD regarding their children’s abilities and challenges in executing a safe road crossing., Additionally, the theme parental concerns and influences on children’s road safety, sheds light on the worries and concerns parents have regarding their children’s pedestrian safety covering the second objective. Finally, the objective of investigating the diverse strategies parents employ to minimize their child’s involvement in pedestrian injuries was addressed in the road safety education: parental views, strategies, and collaboration theme. Although there was some overlap in the experiences shared by the participants, each parent provided a unique perspective and experience that contributed to a more comprehensive picture of the behavior of children with DCD and/or ADHD at the roadside. Importantly, the identified themes were not isolated from one another; they interacted and impacted the overall understanding of pedestrian behavior in this population to tell the everyday story of the parents of children with DCD and/or ADHD.

It is also important to note that the specific challenges and concerns varied depending on whether the child had DCD, ADHD or both DCD and ADHD. For example, parents of children with DCD primarily focused on difficulties with spatial awareness and motor skills, often struggling with judging distances and maneuvering safely around obstacles. Children with ADHD, on the other hand, faced challenges with impulsivity and inattention, which could lead to sudden dashes into traffic or difficulty focusing on potential dangers. Parents of children with co-occurring DCD and ADHD faced a complex interplay of these challenges, requiring constant vigilance and proactive measures to mitigate risks. This highlights the importance of tailoring interventions and support to the specific needs of each group, ensuring effective strategies that address their unique vulnerabilities and promote safe pedestrian behavior.

### Challenges experienced by children at the roadside

The first theme uncovered important insights into the experiences of parents of children with DCD and/or ADHD in relation to roadside behavior and road crossing. Our findings suggest that parents of children with ADHD are concerned about their children’s safety specifically when crossing driveways. Meanwhile, parents of children with DCD were more anxious about complex pedestrian environments like roundabouts. For parents of children with co-occurring DCD and ADHD, the concerns were compounded, encompassing both impulsivity at driveways and difficulties navigating complex environments. Notably, all three groups of parents (children with DCD, children with ADHD, and children with DCD and ADHD) shared a common agreement that zebra crossings, signalized crossings and road crossing patrols are perceived as safer options compared to midblock crossings. This finding emphasizes the significance of structured and controlled crossing sites for enhancing the safety of children with DCD and/or ADHD while navigating roads. This is consistent with previous research indicating that midblock crossings can pose greater risks because of the complexities involved in judging distances, vehicle speeds, walking speeds and making accurate decisions (Purcell et al., 2011: Schwebel et al., 2014).

Regarding roadside behavior, children with ADHD exhibited high levels of activity and energy, which often led to a lack of attention to their surroundings and unintentionally walking or running on the road instead of the pavement. Conversely, a previous study conducted in an experimental setting by Stavrinos et al. (2011) found that children with ADHD-Combined Type demonstrated adequate pavement pedestrian behavior. The differences may be attributed to the inherent limitations of the laboratory setting, which may not fully replicate the complex and dynamic real-life environment characterized by a high volume of sensory stimuli as reported by parents in this study. This can be supported by Öhrström and Skånberg's (2004) exploration of the effects of traffic noise on sleep, where conflicting outcomes between field studies and laboratory experiments were highlighted, indicating potential limitations in the accuracy of results obtained solely from experimental settings. When discussing road crossing behavior, parents of children with ADHD reported their children’s tendency to walk or run across the road, disregarding oncoming vehicles and environmental cues while crossing, which led to an increased possibility of engaging in unsafe crossings. Previous studies support this finding showing that children with ADHD are more likely to engage in unsafe road crossing behaviors, such as crossing when it is not safe, neglecting to look both ways before crossing and running across the road (Clancy et al., 2006; Stavrinos et al., 2011; Wilmut and Purcell, 2020). Furthermore, parents described their child as having “tunnel vision” in which children with ADHD focused solely on their intended destination. However, considering the characteristics associated with ADHD, including inattention and executive dysfunction, the concept of *cognitive tunneling* may provide a more accurate description (Briggs et al., 2016).

On the other hand, children with DCD struggled with spatial awareness and relied heavily on others for guidance, leading to reduced independence and decision-making at the roadside as reported by their parents. Furthermore, parents of children with DCD observed their children when crossing roads visually scanning before crossing but struggling to interpret the visual cues and make appropriate decisions. A study conducted by Purcell et al. (2012) found that children with DCD had lower looming detection thresholds compared to typically developing children, meaning they struggle to recognize an approaching object’s potential threat as quickly as typical children, indicating weaker visual-motor processing skills that could lead to inaccurate crossing decisions. For example, children with DCD might misjudge a vehicles speed and assume a wider available traffic gap, potentially leading to risky situations. Therefore, the parents of children with DCD reported in their interviews that they often relied on other pedestrians or made random decisions, which increased their vulnerability to accidents.

According to parents, children with co-occurring DCD and ADHD displayed a combination of these behavioral characteristics, further increasing their vulnerability at the roadside and crossing roads. The lowered awareness in the DCD and co-occurring groups, but not in the ADHD group, is supported by Loh et al. (2011) who stated that impaired visual–spatial ability may be associated with DCD, while no similar association has been observed with ADHD. Parents spoke about the presence of behaviors attributed to ADHD. Parents of children with both DCD and ADHD reported that they engaged in more risky behavior and displayed significantly less attention compared to those with DCD alone (Wilmut and Purcell, 2020). Thus, the presence of ADHD further exacerbates these difficulties, leading to a potential increase in their vulnerability to accidents.

### Parental concerns and influences on children’s road safety

The second theme captured parental understanding and emotional responses regarding their children’s pedestrian safety, as well as their exploration of potential factors influencing their children’s roadside performance. In this study, parents of children with DCD, ADHD and co-occurring DCD and ADHD expressed concerns about their children’s roadside performance, which aligns with findings from Wilmut and Purcell (2020) who explored the lived experience of adults with DCD and parents of children with DCD using a self-report questionnaire. Brook and Boaz (2006) also found heightened concerns among parents of adolescents with ADHD compared to a typically developing control group. These findings have implications for road safety, as parents may be more inclined to limit their children’s independent community mobility due to their concerns. Previous studies linked independent community mobility to enhanced physical health through active exploration, boosted mental well-being via cognitive development and independent play and stronger social bonds formed through peer interaction and community connection (Pacilli et al., 2013: Qiu and Zhu, 2017). In fact, parents of children with DCD and/or ADHD in this study reported closely monitoring their children’s community movements, limiting active travel while relying on vehicles and limiting unsupervised outings because of these concerns. Moreover, the compromised coordination and communication between parents and children with ADHD when crossing, often exacerbated by anxiety and fear, can directly impact their attention and decision-making (O’Neal et al., 2022). These negative emotions, further heightened by situations where parents and children choose different crossing gaps, can impair children’s ability to process information and make safe choices leading to increased collision risk and unsafe behaviors (O’Neal et al., 2022). Similarly, the emerging evidence indicating the effect of higher task-specific anxiety on motor behavior in children with DCD leading to poor gaze patterns, stepping behavior and gait, could increase the risk of accidents, further emphasizing the potential impact of emotional factors on roadside performance (Harris et al., 2022). Overall, these factors may result in parents adopting an overly protective approach toward their child, potentially leading to increased isolation and delayed development of independent mobility skills. Therefore, parents of children with DCD and/or ADHD were concerned about finding the balance between promoting their children’s independence and prioritizing their safety and well-being as pedestrians.

Furthermore, parents believed that traits related to DCD and/or ADHD seemed to influence their children’s pedestrian performance and road crossing behaviors. Parents of children with ADHD mentioned the influence of mood and temperament on their child’s pedestrian safety. They observed that their child’s impulsiveness, inattention and difficulty concentrating during negative mood episodes can contribute to engaging in unsafe crossing behaviors. Conversely, periods of improved mood and enhanced focus were associated with better performance in pedestrian tasks. Skirrow et al. (2009) conducted a study suggesting that mood instability and ADHD traits may be interconnected and that mood instability could be considered a fundamental aspect of ADHD. However, Clancy et al. (2006) suggested that inattention may have a greater significance in predicting safety in the context of road crossing when compared to a typically developing control group. Further studies found a positive correlation between executive dysfunction and unsafe pedestrian crossings for both children with ADHD and typically developing children (Stavrinos et al., 2011; Tabibi et al., 2022, 2023). Additionally, Tabibi et al. (2023) stated that attentional abilities did not have a substantial impact on determining unsafe behaviors as measured by the Integrated Visual and Auditory Continuous Performance Test (IVA + Plus), a computerized test that assess different components of attention (Sanford and Turner, 1995). Generally, poor pedestrian performance among children with ADHD can collectively be attributed to a combination of ADHD characteristics, such as inattention, impulsivity and lowered concentration, which are influenced by mood and temperament, as well as executive dysfunction leading to the increased risk of unsafe crossing behaviors in children with ADHD. Therefore, the research findings that suggest that a combination of ADHD traits, executive dysfunction and mood and temperament can influence the pedestrian safety of children with ADHD, are supported by parents’ perspectives in this study.

On the other hand, parents of children with DCD believed that their child’s pedestrian performance is related to their inability to judge distance and speed accurately. This aligns with the research on looming sensitivity observed in the study by Purcell et al. (2012), as accurate perceptions of approaching vehicles to avoid collisions requires accurate judgment of the optical equivalent of distance and speed. This is further supported by the finding that children with DCD select insufficient temporal crossing gaps when presented with a virtual task simulating road crossing scenarios across different vehicle approach speeds, indicating difficulties in accurately judging and selecting appropriate gaps for safe crossing (Purcell et al., 2017) and demonstrating deficits in visuomotor adaptation skills (Bo and Lee, 2013). Additionally, this study’s findings indicate that the reported difficulties with body position awareness further contribute to the challenges faced by these children in navigating obstacles and people on the pavement. The presence of fatigue, forgetfulness and diminished self-confidence can further influence the roadside behaviors of children with DCD as reported by parents in the study. Recent research using self-reported questionnaires found a decrease in confidence in road crossing skills among both adults and children with DCD (Wilmut and Purcell, 2020), supporting the findings from this study. However, earlier studies showed no difference in self-reported confidence regarding children aged 6 to 12 years with DCD’s ability to independently and safely cross the road compared to their typically developing peers (Purcell et al., 2012; Purcell and Romijn, 2017). The differences in confidence levels between the current study and studies showing no difference in confidence may be attributed to the older age of participants included in this study. Therefore, developmental changes and the reliance on parent perspectives could contribute to the observed differences in confidence among individuals with DCD. Furthermore, while executive function deficits are also known in DCD (Sartori et al., 2020), their link to pedestrian performance remains unclear despite some associations with poor driving (Kirby et al., 2011). This suggests a potential role of executive functions in pedestrian safety, but more research is needed to understand its specific influence in this context. Overall, the challenges related to spatial awareness and accurate perception experienced by children with DCD could be the main contributors to the elevated likelihood of engaging in unsafe pedestrian behaviors.

For parents of children with co-occurring DCD and ADHD, understanding the underlying causes are more complex due to various factors related to the characteristics of ADHD and DCD. Parents reported that the pedestrian performance of their children can be influenced by inattention and impulsivity, which is related to inhibitory control, in addition to poor perceptual-motor skills and spatial awareness. Previous studies highlighted the presence of overlapping characteristics between DCD and/or ADHD (Bernardi et al., 2015; Harrowell et al., 2018; Wilson et al., 2020; Meachon et al., 2021). For instance, hyperactivity was identified as a co-occurring difficulty among children with DCD (Harrowell et al., 2018). Sartori et al. (2020) also found that children with DCD have poor performance in multiple executive functions including cognitive flexibility, working memory and inhibitory control. However, Meachon et al. (2021) explored the underpinning neurological mechanisms among individuals with DCD and/or ADHD in relation to inhibitory control and found that each group has a distinct executive mechanism despite the overt behavioral similarities. These findings suggest that although individuals with DCD and ADHD may present similar behaviors, including roadside behaviors, their underlying neurological mechanisms are distinct and should be addressed differently.

In summary, parents of children with DCD and/or ADHD in this study are facing the challenge of balancing between fostering children’s independence and ensuring their safety as pedestrians. These parents attributed the current pedestrian performance of their children to the characteristics related to DCD and ADHD. However, emerging evidence indicates that executive dysfunction may serve as the underlying cause of their performance at the roadside. While the results are not conclusive, this implies that the road crossing behaviors of children with DCD and/or ADHD should be approached differently to ensure their road safety or when aiming to develop pedestrian skills. By recognizing the potential influence of executive dysfunction, interventions and strategies tailored to the specific needs of these children could be developed to optimize their road safety and pedestrian abilities.

### Road safety education: parental views, strategies, and collaboration

The third theme explored the approaches adopted by parents and the importance of educational interventions in promoting pedestrian safety among children with DCD and/or ADHD. The findings revealed several key aspects in this domain.

Firstly, parents of children with DCD and/or ADHD expressed concerns about the inadequacy of current pedestrian safety programs in meeting their child’s unique needs. They emphasized the importance of addressing additional needs in preparing their children for the transition to becoming an independent pedestrian through using tailored approaches and multiple modes of delivery. Parents suggested the implementation of customized programs that specifically address the distinctive traits associated with DCD and/or ADHD. For instance, in the case of children with DCD, previous studies identified specific elements that should be considered to enhance pedestrian safety. Notably, research conducted by Purcell et al. (2012) revealed a lower looming detection threshold among children with DCD, which has implications for their ability to perceive and respond to approaching objects or vehicles. Additionally, Purcell and Romijn (2017) suggest that a multimodal approach, involving both allocentric (environment-centered) and egocentric (self-centered) approaches, may be necessary to effectively be used by parents to teach road safety to children with DCD. These findings are closely linked to the reported difficulties in visual processing of perceived information and spatial awareness experienced by children with DCD. Repetition was also reported by parents as an effective element to improve the pedestrian safety of children with DCD and co-occurring DCD and ADHD. For example, parents in this study suggested that simulated environments, which can be virtual or physical, can provide safe repeated opportunities to learn pedestrian skills. Emerging evidence also suggests that virtual reality can be an effective approach for creating a safe environment that facilitates repetitive practice and improves pedestrian safety, benefiting both typically developing children and those with DCD and/or ADHD (Clancy et al., 2006; Purcell and Romijn, 2017; Morrongiello et al., 2018; Schwebel et al., 2018). By incorporating these insights and strategies into tailored programs, the specific challenges faced by children with DCD and/or ADHD could be effectively addressed.

Secondly, parents of children with DCD and/or ADHD implemented various strategies to enhance their children’s pedestrian safety and decrease the risk of road crossing injuries. Despite efforts to provide pedestrian safety training, the alarming rate of fatalities among children on our roads remains a significant cause for concern and it is evident that implementing behavioral strategies can be cost-efficient and play a crucial role in enhancing safety (Schwebel et al., 2014). Furthermore, adopting these strategies to incorporate the distinct requirements of children can be effective in fostering their pedestrian skills and formulating tailored interventions. Therefore, it is imperative to understand the strategies used by parents to mitigate the risk of child pedestrian injuries. A summary of the parent strategies identified by each group is provided in Table 4. Common strategies used by parents of children with DCD and/or ADHD include constant forward planning, selecting quieter roads, driving to school, using verbal prompts, crossing with peers, walking in the middle of a group, repeated practice, avoiding traffic, using hand gestures, offering physical guidance and fostering familiarity with the environment. However, it is important to note that some of these strategies may not be feasible for all parents, depending on their individual circumstances. It is also worth considering that some of these strategies, while prioritizing pedestrian safety, may hinder the development of road crossing skills necessary for future independent mobility. For example, parents who consistently drive their children to school may not be able to provide an opportunity for their children to practice crossing the road safely in an unsupervised environment. Therefore, it is important for parents to identify strategies that are suitable for their children and their circumstances, while also considering the long-term impact of these strategies.

Moreover, the responsibility of ensuring pedestrian safety for children with DCD and/or ADHD extends beyond parents alone and involves collaboration among schools and local authorities. While parents of children with DCD and/or ADHD in this study face time constraints, they actively take on the responsibility of teaching their children pedestrian skills and ensuring their safety on the roads. These parental efforts align with previous research (Morrongiello and Corbett, 2015; Ngu et al., 2016; Zare et al., 2019), which underscores the crucial role of parental involvement in enhancing pedestrian skills and mitigating the risk of accidents. Additionally, collaborative efforts involving schools and local authorities showed effective results in promoting road safety education. Studies investigating the impact of road crossing programs involving the active participation of schoolteachers and police officers in enhancing pedestrian skills (Schwebel et al., 2018; Zare et al., 2018; McLaughlin et al., 2019; Jiang et al., 2021) show the valuable contribution of educational institutions and local authorities in fostering pedestrian skills among typically developing children. Additional research is needed to further explore the collaborative efforts between these stakeholders specifically focusing on children with DCD and/or ADHD. Another crucial obstacle to consider when promoting pedestrian safety training for children with DCD, in particular, is the lack of widespread awareness and knowledge about the condition, even among medical and educational professionals (Hunt et al., 2021; Meachon et al., 2023). Therefore, fostering collaborative efforts must include raising awareness and providing targeted training for educators, healthcare professionals and local authorities, to equip them with the knowledge and skills necessary to support children with DCD as well as the other two groups. By recognizing the shared responsibility and fostering collaboration among parents, schools and local authorities, comprehensive and effective measures could be implemented to promote the pedestrian safety of children with ADHD and/or DCD.

The study provides findings into the lived experience of parents of children with DCD and/or ADHD in the context of road crossing. However, there are certain limitations to consider. One limitation to this study is that the sample size was relatively small, with only 14 participants, which may limit the variability and diversity of perspectives represented. Furthermore, the limited subgroup size of only three participants with DCD presents a specific challenge. Given the known complexity and heterogeneity of DCD, this small sample may not adequately capture the full range of experiences and challenges faced by this group within the context of road crossing. This limits the study’s ability to confidently generalize findings to the broader population of parents of children with DCD and may mask potentially specific perspectives or concerns unique to this subgroup. Moreover, while the study aimed to understand parental perspectives across different age groups, the age ranges within each group varied slightly (DCD: 10–17 years, ADHD: 7–13 years, Co-occurring: 7–16 years). This variation may have influenced parental perspectives of age-related differences in pedestrian behaviors and limits the ability to draw qualitatively based conclusions about age-based trends from the current dataset. Nonetheless, the study offers valuable preliminary findings that warrant further investigation. Moreover, the study focused solely on parents’ perspectives regarding road crossing, overlooking the child’s perspective and the broader experiences and challenges parents face in other aspects of parenting. Future research should explore the perspectives of children with DCD and/or ADHD regarding road crossing and investigate additional dimensions of parenting challenges and examine the impact of DCD and/or ADHD on other daily activities beyond pedestrian safety. Additionally, it is worth noting that the semi-structured interviews were conducted online, potentially excluding individuals without internet access or those less comfortable with online communication. This could introduce bias, as those with different access or preferences may possess unique perspectives or experiences related to road crossing.

In conclusion, the findings revealed several key insights that represent parents’ perspectives of children with DCD and/or ADHD regarding the pedestrian risks faced by their children. Firstly, the importance of structured and controlled pedestrian crossing sites was emphasized by parents. Secondly, parents expressed heightened concerns about their children’s performance and safety at the roadside, leading to increased monitoring and a more protective approach. Addressing these concerns is essential to promote the independence and well-being of these children. Additionally, while the underlying causes are not yet fully understood, it is evident that the reported road crossing behaviors of children with DCD and/or ADHD require a distinct approach to better develop their pedestrian skills effectively. Furthermore, parents implemented various strategies to mitigate the risks associated with roadside activities, but it is important to balance independence and the development of pedestrian skills. Lastly, promoting pedestrian safety for children with DCD and/or ADHD will require collaboration and shared responsibility between parents, schools and local authorities to implement comprehensive measures to ensure their safety and well-being. These findings contribute to the understanding of the perspectives of parents and provide valuable guidance for the development of targeted interventions and policies to promote the road safety of children with DCD and/or ADHD.

## Data availability statement

The original contributions presented in the study are included in the article/supplementary material, further inquiries can be directed to the corresponding author.

## Ethics statement

The studies involving humans were approved by the School of Healthcare Sciences Research Ethics Committee. The studies were conducted in accordance with the local legislation and institutional requirements. The participants provided their written informed consent to participate in this study.

## Author contributions

RF: Writing – review & editing, Writing – original draft, Visualization, Methodology, Investigation, Formal Analysis, Data curation, Conceptualization. KW: Writing – review & editing, Validation, Supervision. HH: Writing – review & editing, Validation, Supervision. CP: Conceptualization, Writing – review & editing, Validation, Supervision, Methodology.

## References

[ref1] Abdelnour E. Jansen M. O. Gold J. A. (2022). ADHD diagnostic trends: increased recognition or Overdiagnosis? Mo. Med. 119, 467–473. PMID: 36337990 PMC9616454

[ref9001] American Psychiatric Association. (2013). Diagnostic and statistical manual of mental disorders (5th ed.). Arlington, VA: Author.

[ref9002] Arigo D. Pagoto S. Carter-Harris L. Lillie S. E. Nebeker C. (2018). Using social media for health research: methodological and ethical considerations for recruitment and intervention delivery. Digit. Health. 4:2055207618771757. doi: 10.1177/2055207618771757 29942634 PMC6016568

[ref9003] Bender J. L. Cyr A. B. Arbuckle L. Ferris L. E. (2017). Ethics and privacy implications of using the internet and social media to recruit participants for health research: a privacy-by-design framework for online recruitment. J. Med. Internet Res. 19:e104. doi: 10.2196/jmir.7029 28385682 PMC5399223

[ref2] Bernardi M. Leonard H. C. Hill E. L. Henry L. A. (2015). Brief report: response inhibition and processing speed in children with motor difficulties and developmental coordination disorder. Child Neuropsychol. 22, 627–634. doi: 10.1080/09297049.2015.1014898, PMID: 25761255

[ref3] Blank R. Barnett A. L. Cairney J. Green D. Kirby A. Polatajko H.. (2019). International clinical practice recommendations on the definition, diagnosis, assessment, intervention, and psychosocial aspects of developmental coordination disorder. Dev. Med. Child Neurol. 61, 242–285. doi: 10.1111/dmcn.14132, PMID: 30671947 PMC6850610

[ref4] Bo J. Lee C. M. (2013). Motor skill learning in children with developmental coordination disorder. Res. Dev. Disabil. 34, 2047–2055. doi: 10.1016/j.ridd.2013.03.012 23584185

[ref6] Braun V. Clarke V. (2022). Conceptual and design thinking for thematic analysis. Qual. Psychol. 9, 3–26. doi: 10.1037/qup0000196

[ref7] Braun V. Clarke V. (2023). Toward good practice in thematic analysis: avoiding common problems and be (com) ing a knowing researcher. Int. J. Transgender Health 24, 1–6. doi: 10.1080/26895269.2022.2129597, PMID: 36713144 PMC9879167

[ref8] Briggs G. F. Hole G. J. Land M. F. (2016). Imagery-inducing distraction leads to cognitive tunnelling and deteriorated driving performance. Transport. Res. F: Traffic Psychol. Behav. 38, 106–117. doi: 10.1016/j.trf.2016.01.007

[ref9] Brook U. Boaz M. (2006). Adolescents with attention deficit and hyperactivity disorder/learning disability and their proneness to accidents. Indian J. Pediatr. 73, 299–303. doi: 10.1007/BF02825823, PMID: 16816490

[ref10] Bussing R. Fernandez M. Harwood M. Hou W. Garvan C. Eyberg S.. (2008). Parent and teacher SNAP-IV ratings of attention deficit hyperactivity disorder symptoms. Assessment 15, 317–328. doi: 10.1177/1073191107313888, PMID: 18310593 PMC3623293

[ref11] Clancy T. A. Rucklidge J. J. Owen D. (2006). Road-crossing safety in virtual reality: a comparison of adolescents with and without ADHD. J. Clin. Child Adolesc. Psychol. 35, 203–215. doi: 10.1207/s15374424jccp3502_4 16597216

[ref12] Department for Transport. (2019). Reported road casualties in Great Britain: 2018 annual report. Available at: https://assets.publishing.service.gov.uk/government/uploads/system/uploads/attachment_data/file/834585/reported-road-casualties-annual-report-2018.pdf (Accessed June 24, 2023).

[ref13] Department for Transport. (2020). Reported road casualties in Great Britain: 2019 annual report. Available at: https://assets.publishing.service.gov.uk/government/uploads/system/uploads/attachment_data/file/922717/reported-road-casualties-annual-report-2019.pdf (Accessed June 24, 2023).

[ref14] Etikan I. Musa S. A. Alkassim R. S. (2016). Comparison of convenience sampling and purposive sampling. Am. J. Theor. Appl. Stat. 5, 1–4. doi: 10.11648/j.ajtas.20160501.11

[ref9004] Gau S. S. F. Shang C. Y. Liu S. K. Lin C. H. Swanson J. M. Liu Y. C.. (2008). Psychometric properties of the Chinese version of the Swanson, Nolan, and Pelham, version IV scale–parent form. Int J Methods Psychiatr Res, 17, 35–44. doi: 10.1002/mpr.237 18286459 PMC6878250

[ref16] Goulardins J. B. Rigoli D. Licari M. Piek J. P. Hasue R. H. Oosterlaan J.. (2015). Attention deficit hyperactivity disorder and developmental coordination disorder: two separate disorders or do they share a common etiology. Behav. Brain Res. 292, 484–492. doi: 10.1016/j.bbr.2015.07.009, PMID: 26168770

[ref17] Hall C. L. Guo B. Valentine A. Z. Groom M. J. Daley D. Sayal K.. (2020). The validity of the SNAP-IV in children displaying ADHD symptoms. Assessment 27, 1258–1271. doi: 10.1177/1073191119842255, PMID: 30991820

[ref18] Harris S. Purcell C. Wilmut K. (2022). Moving with confidence: how does anxiety impede performance in individuals with developmental coordination disorder (DCD)? Curr. Dev. Disord. Rep. 9, 98–104. doi: 10.1007/s40474-022-00251-7

[ref19] Harrowell I. Hollén L. Lingam R. Emond A. (2018). The impact of developmental coordination disorder on educational achievement in secondary school. Res. Dev. Disabil. 72, 13–22. doi: 10.1016/j.ridd.2017.10.014, PMID: 29080482 PMC5770330

[ref20] Holloway I. Todres L. (2003). The status of method: flexibility, consistency and coherence. Qual. Res. 3, 345–357. doi: 10.1177/1468794103033004

[ref21] Hunt J. Zwicker J. G. Godecke E. Raynor A. (2021). Awareness and knowledge of developmental coordination disorder: a survey of caregivers, teachers, allied health professionals and medical professionals in Australia. Child Care Health Dev. 47, 174–183. doi: 10.1111/cch.12824, PMID: 33140459 PMC7894302

[ref22] Jiang K. Wang Y. Feng Z. Cui J. Huang Z. Yu Z.. (2021). Research on intervention methods for children’s street-crossing behaviour: application and expansion of the theory of “behaviour spectrums”. Accid. Anal. Prev. 152:105979. doi: 10.1016/j.aap.2021.105979, PMID: 33548586

[ref23] Kirby A. Sugden D. Edwards L. (2011). Driving behaviour in young adults with developmental co-ordination disorder. J. Adult Dev. 18, 122–129. doi: 10.1007/s10804-011-9120-4

[ref24] Kirby A. Williams N. Thomas M. Hill E. L. (2013). Self-reported mood, general health, wellbeing and employment status in adults with suspected DCD. Res. Dev. Disabil. 34, 1357–1364. doi: 10.1016/j.ridd.2013.01.003, PMID: 23417140

[ref26] Loh P. R. Piek J. P. Barrett N. C. (2011). Comorbid ADHD and DCD: examining cognitive functions using the WISC-IV. Res. Dev. Disabil. 32, 1260–1269. doi: 10.1016/j.ridd.2011.02.008, PMID: 21377321

[ref28] McLaughlin C. M. Barry W. E. Barin E. N. Mert M. Lowery C. Upperman J. S.. (2019). Interactive education is associated with lower incidence of pedestrian-related injury in children. J. Surg. Res. 244, 57–62. doi: 10.1016/j.jss.2019.06.015, PMID: 31279264 PMC6815706

[ref29] Meachon E. J. Melching H. Alpers G. W. (2023). The overlooked disorder: (un) awareness of developmental coordination disorder across clinical professions. Adv. Neurodev. Disord., 1–9. doi: 10.1007/s41252-023-00334-5

[ref30] Meachon E. J. Meyer M. Wilmut K. Zemp M. Alpers G. W. (2021). Evoked potentials differentiate developmental coordination disorder from attention-deficit/hyperactivity disorder in a stop-signal task: a pilot study. Front. Hum. Neurosci. 15:629479. doi: 10.3389/fnhum.2021.629479, PMID: 33776670 PMC7990764

[ref31] Morrongiello B. A. Corbett M. (2015). Using a virtual environment to study child pedestrian behaviours: a comparison of parents’ expectations and children's street crossing behaviour. Inj. Prev. 21, 291–295. doi: 10.1136/injuryprev-2014-041508, PMID: 25825352

[ref32] Morrongiello B. A. Corbett M. Beer J. Koutsoulianos S. (2018). A pilot randomized controlled trial testing the effectiveness of a pedestrian training program that teaches children where and how to cross the street safely. J. Pediatr. Psychol. 43, 1147–1159. doi: 10.1093/jpepsy/jsy056, PMID: 30113643 PMC6199176

[ref34] Ngu L. S. Hanafi Z. Taslikhan M. (2016). Influence of parental involvement on academic achievement. Int. J. Adv. Educ. Res. 1, 1–4.

[ref35] O’Neal E. E. Rahimian P. Jiang Y. Zhou S. Nikolas M. Kearney J. K.. (2022). How do child ADHD symptoms and Oppositionality impact parent–child interactions when crossing virtual roads? J. Pediatr. Psychol. 47, 337–349. doi: 10.1093/jpepsy/jsab102, PMID: 34664654

[ref36] Öhrström E. Skånberg A. (2004). Sleep disturbances from road traffic and ventilation noise—laboratory and field experiments. J. Sound Vib. 271, 279–296. doi: 10.1016/S0022-460X(03)00753-3

[ref37] Pacilli M. Giovannelli I. Prezza M. Augimeri M. (2013). Children and the public realm: antecedents and consequences of independent mobility in a group of 11–13-year-old Italian children. Child. Geogr. 11, 377–393. doi: 10.1080/14733285.2013.812277

[ref38] Parr M. N. Tang H. Mallaro S. R. Kearney J. K. Plumert J. M. (2021). Do inattention/hyperactivity and motor timing predict Children’s virtual road-crossing performance? J. Pediatr. Psychol. 46, 1130–1139. doi: 10.1093/jpepsy/jsab054, PMID: 34402519

[ref39] Purcell C. Romijn A. R. (2017). Appropriateness of different pedagogical approaches to road safety education for children with developmental coordination disorder (DCD). Res. Dev. Disabil. 70, 85–93. doi: 10.1016/j.ridd.2017.08.010, PMID: 28918308

[ref40] Purcell C. Wann J. P. Wilmut K. Poulter D. (2011). Roadside judgments in children with developmental co-ordination disorder. Res. Dev. Disabil. 32, 1283–1292. doi: 10.1016/j.ridd.2010.12.022, PMID: 21247732

[ref41] Purcell C. Wann J. P. Wilmut K. Poulter D. (2012). Reduced looming sensitivity in primary school children with developmental co-ordination disorder. Dev. Sci. 15, 299–306. doi: 10.1111/j.1467-7687.2011.01123.x, PMID: 22490171

[ref42] Purcell C. Wilmut K. Wann J. P. (2017). The use of visually guided behaviour in children with developmental coordination disorder (DCD) when crossing a virtual road. Hum. Mov. Sci. 53, 37–44. doi: 10.1016/j.humov.2016.11.007, PMID: 27939726

[ref43] Qiu L. Zhu X. (2017). Impacts of housing and community environments on children’s independent mobility: a systematic literature review. New ARCH 18:43. doi: 10.14621/tna.20170205

[ref44] Ramos-Quiroga J. A. Daigre C. Valero S. Bosch R. Gómez-Barros N. Nogueira M.. (2009). Validation of the Spanish version of the attention deficit hyperactivity disorder adult screening scale (ASRS v. 1.1): a novel scoring strategy. Rev. Neurol. 48, 449–452. 19396760

[ref45] Sanford J. A. Turner A. (1995). Manual for the integrated visual and auditory continuous performance test. Richmond, VA: BrainTrain.

[ref46] Sartori R. F. Valentini N. C. Fonseca R. P. (2020). Executive function in children with and without developmental coordination disorder: a comparative study. Child Care Health Dev. 46, 294–302. doi: 10.1111/cch.12734, PMID: 31845379

[ref47] Schwebel D. C. Barton B. K. Shen J. Wells H. L. Bogar A. Heath G.. (2014). Systematic review and meta-analysis of behavioral interventions to improve child pedestrian safety. J. Pediatr. Psychol. 39, 826–845. doi: 10.1093/jpepsy/jsu024, PMID: 24864275 PMC4138804

[ref48] Schwebel D. C. Wu Y. Li P. Severson J. He Y. Xiang H.. (2018). Featured article: evaluating smartphone-based virtual reality to improve Chinese schoolchildren’s pedestrian safety: a non-randomized trial. J. Pediatr. Psychol. 43, 473–484. doi: 10.1093/jpepsy/jsx147, PMID: 29216384 PMC5961228

[ref49] Scott T. L. Tulloch K. (2021). Is community mobility contingent upon driving? Attitudes toward and intentions to use alternative modes of transport according to a mixed-aged sample. J. Transp. Health 20:100974. doi: 10.1016/j.jth.2020.100974

[ref50] Shaw B. Bicket M. Elliott B. Fagan-Watson B. Mocca E. Hillman M. (2015). Children’s independent mobility: an international comparison and recommendations for action. London: Policy Studies Institute.

[ref51] Skirrow C. McLoughlin G. Kuntsi J. Asherson P. (2009). Behavioral, neurocognitive and treatment overlap between attention-deficit/hyperactivity disorder and mood instability. Expert. Rev. Neurother. 9, 489–503. doi: 10.1586/ern.09.2, PMID: 19344301

[ref52] Stav W. B. (2014). Updated systematic review on older adult community mobility and driver licensing policies. Am. J. Occup. Ther. 68, 681–689. doi: 10.5014/ajot.2014.011510, PMID: 25397763

[ref53] Stavrinos D. Biasini F. J. Fine P. R. Hodgens J. B. Khatri S. Mrug S.. (2011). Mediating factors associated with pedestrian injury in children with attention-deficit/hyperactivity disorder. Pediatrics 128, 296–302. doi: 10.1542/peds.2010-3829, PMID: 21788213

[ref54] Tabibi Z. Schwebel D. C. Juzdani M. H. (2023). How does attention deficit hyperactivity disorder affect children’s road-crossing? A case-control study. Traffic Inj. Prev. 24, 315–320. doi: 10.1080/15389588.2023.2181664, PMID: 36867075 PMC10400341

[ref55] Tabibi Z. Schwebel D. C. Zolfaghari H. (2022). Road-crossing behavior in complex traffic situations: a comparison of children with and without ADHD. Child Psychiatry Hum. Dev. 53, 1186–1193. doi: 10.1007/s10578-021-01200-y, PMID: 34106381 PMC10404361

[ref56] Tapiro H. Meir A. Parmet Y. Oron-Gilad T. (2014). Visual search strategies of child-pedestrians in road crossing tasks. In Proceedings of the Human Factors and Ergonomics Society Europe, 119–130.

[ref57] Thomas F. B. (2022). The role of purposive sampling technique as a tool for informal choices in a social sciences in research methods. Just Agric. 2, 1–8.

[ref9005] Topolovec-Vranic J. Natarajan K. (2016). The use of social media in recruitment for medical research studies: a scoping review. J. Med. Internet Res. 18:e286. doi: 10.2196/jmir.5698 27821383 PMC5118584

[ref59] Wilmut K. Purcell C. (2020). The lived experience of crossing the road when you have developmental coordination disorder (DCD): the perspectives of parents of children with DCD and adults with DCD. Front. Psychol. 11:587042. doi: 10.3389/fpsyg.2020.587042, PMID: 33329244 PMC7710519

[ref60] Wilmut K. Purcell C. (2021). The nature of the risk faced by pedestrians with neurodevelopmental disorders: a systematic review. Accid. Anal. Prev. 149:105886. doi: 10.1016/j.aap.2020.105886, PMID: 33248701

[ref9006] Wilson B. N. Crawford S. G. Green D. Roberts G. Aylott A. Kaplan B. J. (2009). Psychometric properties of the revised developmental coordination disorder questionnaire. Phys. Occup. Ther. Pediatr. 29, 182–202. doi: 10.1080/01942630902784761 19401931

[ref61] Wilson B. N. Crawford S. G. Roberts G., (2007). The developmental coordination disorder questionnaire 2007 (DCDQ’07). Administrative manual for the DCDQ107 with psychometric properties, 267–272. Available at: http://dcdq.ca/ (Accessed February 22, 2023).

[ref62] Wilson P. Ruddock S. Rahimi-Golkhandan S. Piek J. Sugden D. Green D.. (2020). Cognitive and motor function in developmental coordination disorder. Dev. Med. Child Neurol. 62, 1317–1323. doi: 10.1111/dmcn.14646 32770756

[ref63] World Health Organization. (2018). Disease, injury and causes of death country estimates, 2000–2015. Available at: http://www.who.int/healthinfo/global_burden_disease/estimates_country_2000_2015/en/ (Accessed June 04, 2023).

[ref64] Zare H. Niknami S. Heidarnia A. Fallah M. H. (2018). Improving safe street-crossing behaviors among primary school students: a randomized controlled trial. Health Promot. Perspect. 8, 308–314. doi: 10.15171/hpp.2018.44, PMID: 30479986 PMC6249492

[ref9007] Zare H. Niknami S. Heidarnia A. Fallah M. H. (2019). Traffic safety education for child pedestrians: A randomized controlled trial with active learning approach to develop street-crossing behaviors. Transportation research part F: traffic psychology and behaviour, 60, 734–742. doi: 10.1016/j.trf.2018.10.021

