# Supplementary material for: Roadside experiences of parents of children with developmental coordination disorder and/or attention deficit hyperactivity disorder
Source: Front Hum Neurosci. 2024 Apr 10;18:1339043. doi: 10.3389/fnhum.2024.1339043 (PMC11039856; doi:10.3389/fnhum.2024.1339043)
Supplement: Supplementary file 1 [file Table_1.DOCX]

Semi-structured interview questions

# Questions

1. To start, I would like you to walk me through your normal day while you’re walking around the community with your child? What does it look like?
2. Can you describe the physical environment that you typically walk around everyday such as streets and type of road crossings?
3. Can you tell me more about your child’s behaviour at the roadside and when crossing a road?
4. How do you feel about your child’s performance at the roadside and when crossing a road? Why?
5. What could be the reason for this roadside behaviour?
6. What do you think would help your child to increase his/her safety at the roadside?
7. Do you think ADHD and/or DCD medication makes any difference to your child’s performance at the roadside?
8. Has your child completed any road safety education at school? If so do you remember what this involved and whether it made a difference to your child’s road safety behaviour ?
9. What do you think an effective training programme might include for your child? Why?

1. Who do you think should deliver road crossing training? Why?
2. Can you tell me how you deal with any risk at the roadside when you are with your child?
